# Biofilm exacerbates antibiotic resistance: Is this a current oversight in antimicrobial stewardship?

**DOI:** 10.1186/s13756-020-00830-6

**Published:** 2020-10-20

**Authors:** Philip Bowler, Christine Murphy, Randall Wolcott

**Affiliations:** 1 Infection Prevention and Control, ConvaTec Ltd, Deeside, UK; 2 The Ottawa Hospital Limb Preservation Centre, Ottawa, Canada; 3 Southwest Regional Wound Care Center, Lubbock, TX USA

**Keywords:** Biofilm, Environment, Antibiotic resistance, Antibiotic tolerance, Antimicrobial stewardship

## Abstract

**Objective:**

To raise awareness of the role of environmental biofilm in the emergence and spread of antibiotic resistance and its consideration in antimicrobial stewardship.

**Background:**

Antibiotic resistance is a major threat to public health. Overuse of antibiotics, increased international travel, and genetic promiscuity amongst bacteria have contributed to antibiotic resistance, and global containment efforts have so far met with limited success. Antibiotic resistance is a natural mechanism by which bacteria have adapted to environmental threats over billions of years and is caused either by genetic mutations or by horizontal gene transfer. Another ancient survival strategy involves bacteria existing within a self-produced polymeric matrix, which today is termed biofilm. Biofilm similarly enables bacterial tolerance to environmental threats, and also encourages the transfer of antibiotic resistance genes between bacterial species. This natural and ubiquitous mode of bacterial life has not been considered amongst strategies to tackle antibiotic resistance in healthcare facilities, despite its ability to significantly enhance bacterial survival and persistence, and to encourage antibiotic resistance.

**Conclusion:**

Biofilm must be considered synonymously with antibiotic resistance because of its proficiency in transferring resistance genes as well as its innate phenotypic tolerance to antibiotics. Although biofilm falls outside of the current definition of antimicrobial stewardship, greater awareness of the existence, ubiquity, and consequences of environmental biofilm amongst healthcare practitioners is crucial to improving hygiene practices and controlling the emergence and spread of antibiotic resistance in healthcare facilities.

## Antibiotic resistance

Resistance to antibiotics, through either genomic mutation or transfer of resistance-conferring genes between bacterial species [1], is one of the most significant, challenging and urgent threats to global public health today. The surge in antibiotic resistance in recent decades is primarily attributed to the dramatic increase in global travel [2], excessive and inappropriate use of antibiotics in both human and animal welfare, over-the-counter availability of antibiotics, poor sanitation and hygiene, and the environmental recycling of non-metabolised antibiotics through human and animal consumption [3]. Given the fact that no new class of antibiotic has received regulatory approval since the late 1980s [4], greater understanding of biofilm and its association with antibiotic resistance and tolerance is essential to preserve and prolong the therapeutic value of existing antibiotics.

Despite continuing efforts over the last decade from global health organisations such as the US Centers for Disease Control and Prevention (CDC), the UK Department of Health (DoH), and the World Health Organisation (WHO) to introduce guidelines for the appropriate and responsible use of antibiotics, antibiotic resistance remains a growing problem [3, 5]. In primary care in England, inappropriate antibiotic prescribing of up to 23% has been reported [6]. With continuing poor awareness amongst the public, policymakers and even health care professionals, significant challenges to the promotion of responsible antibiotic use remain.

In 2018, the Organisation for Economic Co-operation and Development (OECD), published an extensive report (“Stemming the Superbug Tide—just a few dollars more”), at the request of its Member countries (spanning North and South America, Europe and Asia–Pacific regions) to consider actions necessary to prevent the emergence and spread of antimicrobial resistance (AMR), and to help governments implement them [7]. The comprehensive report concluded that the burden of AMR on public health and healthcare expenditure could be drastically reduced by promoting better hygiene, ending over-prescription of antibiotics, rapid testing for patients to confirm bacterial infections, delays in prescribing antibiotics, and mass media campaigns [7]. Although the OECD analysis has provided the most comprehensive and detailed assessment of the health and economic impact of AMR to-date, the conclusions for controlling AMR are similar to those reported by other organisations such as the CDC and the WHO. Importantly, one factor that is inextricably linked to, and exacerbates antibiotic resistance has been consistently overlooked to date; this factor is biofilm.

Since the mid-1990’s, the term ‘antimicrobial stewardship’ has been increasingly used as a vehicle for promoting the careful and responsible use of antimicrobial agents [8]. The primary focus has been on facilitating the sustainability and continued effectiveness of antibiotics in treating serious infections. However, given the close and evolutionary link between antibiotic resistance and biofilm, it is perhaps prudent to promote greater awareness of the implications of biofilm amongst the healthcare profession.

## Antibiotic tolerance

Tolerance has been defined as the ability of bacteria to survive antibiotic exposure without developing resistance [9]. Tolerance has also been reported to invariably precede antibiotic resistance, which indicates that preventing tolerance may offer new insight into controlling antibiotic resistance [9]. Whereas antibiotic resistance is genetically induced via either mutations or horizontal gene transfer, antibiotic tolerance involves bacterial survival via dormant persister cell [10] and biofilm phenotypic states [11]. Antibiotics that act on actively metabolising cells (e.g. cell wall synthesis) often fail in the presence of non-metabolising (dormant and/or biofilm-associated) bacterial populations, and this tolerance mechanism has been associated with persistent, chronic infections [12, 13].

## The biofilm factor

Bacteria naturally and preferentially live as communities attached to a surface. Once attached, bacterial cells establish and organise themselves within a self-produced extracellular polymeric substance (EPS) to form a matrix that provides protection from environmental threats, thereby providing an extremely effective survival strategy. The term ‘biofilm’ was first used to describe this predominant form of bacterial life in environmental microbiology in 1935, and from 1985 it became commonplace in medical microbiology [14]. Although ‘biofilm’ is recent in name, it is the oldest life-form on Earth [15], and it has recently been reported that biofilms dominate all habitats on the Earth’s surface, accounting for up to 80% of the approximate 1.2 × 10^30^ bacterial cell population [16]. In 1978, Bill Costerton, an eminent microbiologist in the field, and co-workers published a paper in *Scientific American* titled ‘How bacteria stick’ [17]. At the time, biofilm was referred to as a ‘glycocalyx’ that tenaciously adhered bacteria to surfaces ranging from teeth and lungs, to rocks submerged in fast-flowing streams. Costerton et al. [17] concluded that if adhesion played a significant role in the success of pathogenic bacteria, then the prevention of adhesion could be an effective way to combat infection.

The EPS biofilm matrix enables communities of bacteria to exist in close proximity [18] and provides an ideal reservoir for the cellular exchange of plasmids encoding for resistance to antibiotics, thus potentially promoting the spread of bacterial resistance [19]. Horizontal transfer of resistance-conferring genes between bacterial cells within biofilm and has been reported as being 700 times more efficient than among free-living, planktonic bacterial cells [20].

Biofilm is a primary cause of chronic infections such as otitis media, those associated with indwelling medical devices (e.g. catheters, infected surgical implants), infections associated with cystic fibrosis, osteomyelitis, rhinosinusitis, and wound infections. Non-healing wounds in particular, are characterised by complex and mixed bacterial populations, often involving antibiotic-resistant bacteria as well as phenotypically tolerant bacteria in biofilm form [21, 22]. This is an extremely daunting clinical scenario in terms of both therapeutic outcomes and bacterial plasmid promiscuity. By physically disrupting wound biofilm in vivo, Wolcott et al. [23] identified a 24–48 h therapeutic window during which antibiotic therapy was more effective. This indicates that strategies designed to disrupt biofilm can facilitate antimicrobial effectiveness. The European Society of Clinical Microbiology and Infectious Diseases (ESCMID) 2014 guidelines on biofilm diagnosis and treatment, identified the need for ‘new combinations of antibiotics with biofilm-dissolving drugs’ [24], and this is increasingly acknowledged as a necessary approach to controlling biofilm and facilitating antimicrobial effectiveness. Recently, bacteriophage used in combination with ciprofloxacin was shown to be effective in treating *Pseudomonas aeruginosa* biofilm infection in cystic fibrosis subjects [25]. A wound dressing containing a combination of anti-biofilm and antimicrobial agents has been shown to promote healing in previously recalcitrant, biofilm-impeded wounds [26].

The biofilm factor is clearly of considerable clinical importance: it protects bacteria from antimicrobial agents leading to persistent and difficult to treat chronic infections, and it exacerbates the spread of antibiotic resistance.

## Biofilm in healthcare facilities

Biofilms thrive in moist environments and have been reported to be involved in ~ 65% of nosocomial infections including major hospital acquired infections such as those associated with indwelling catheters and prostheses. Biofilm can reside on fomites such as sink drains and taps [27]. An antibiotic-resistant strain of *Klebsiella pneumoniae* was reported to efficiently transfer its extended-spectrum β-lactamase-encoding plasmid amongst bacteria within biofilm, and survive in a hospital environment [28]. A study conducted in German hospitals with high antibiotic consumption regularly detected antibiotic residues in toilets, sink siphons and shower drains [29]. Subsequent flushing of wastewater was successful in removing antibiotic residues, but after temporal stagnation, antibiotics were again detected [29]. This confirms the ability of biofilm to rapidly reform on wet surfaces, and act as a reservoir for accumulation and reoccurrence of antibiotics in hospital sanitary units [29]. The combination of high antibiotic use and environmental biofilms in intensive care units has been recognised as a mechanism enabling increased genetic exchange amongst associated bacteria, leading to persistence of antibiotic-resistant bacteria despite terminal cleaning [30]. Dispersal of carbapenem-resistant Enterobacteriaceae from sink units has also been observed, particularly if sink design facilitates biofilm formation within the drain [31].

With increasing awareness of the ubiquity and implications of environmental biofilm in healthcare facilities, efforts to implement biofilm control measures are emerging. Engineering interventions (e.g. tap outlets and sink drain design), heat application, electromechanical vibration, and use of more potent anti-biofilm agents (e.g. acetic acid, oxidising agents) have all shown some success in removing environmental biofilm and facilitating infection control [32–34]. Additionally, sanitising agents based on non-pathogenic probiotic bacteria (*Bacillus* spp.) have recently been shown to inhibit hospital-associated pathogens, while also displaying high genetic stability with no evidence of acquisition of antibiotic resistance genes [35]. Given the significantly increased tolerance of biofilm bacteria compared with their planktonic counterparts [34], strategies to disrupt and remove biofilm, enhance activity of biocides against disrupted biofilm cells, and prevent the re-establishment of biofilm are imperative. This is likely to involve a multifaceted hygiene protocol that includes physical methods to remove established biofilm from surfaces, detergents and chelating agents [27], and possibly biological agents (probiotics) [35] to facilitate detachment and break-up of biofilm matrix, and the use of biocides to kill associated exposed bacteria. Efforts are also being made to alter the surface properties of medical devices and equipment (e.g. hydrophilic surfaces), and incorporate antimicrobial agents (e.g. copper, probiotics) into surfaces that help to prevent bacterial attachment and reduce transmission of pathogens in the clinical environment [27, 35, 36]. Stopping bacteria from sticking, as quoted by Costerton back in 1978, is clearly an essential component of infection control practices in healthcare facilities [17].

Although biofilm is known to thrive in moist environments, the biofilm threat is intensified by the more recent awareness of the existence of ‘dry surface biofilm’ (DSB) [37]. Hospital surfaces can no longer be considered as being ‘inanimate’ [38]. In a recent study, polymicrobial biofilm containing multidrug-resistant organisms (MDROs) was shown to persist for up to 12 months on equipment and furnishings in an intensive care unit, despite prior terminal cleansing involving detergent and bleach [39]. Polymicrobial DSBs were recovered from 95% of 61 terminally-cleaned items (e.g. keyboards, patient folders, hand sanitising bottles) from three UK hospitals, indicating the ubiquity of bacterial pathogens on hospital surfaces, despite regular cleaning and disinfection [37]. Other studies have similarly detected DSBs on hospital items including clothing, curtains, bed linen, neckties, library books, stethoscopes and cell phones [40–42]. Transfer of DSB by hands to multiple fomites, has also been demonstrated, again demonstrating the necessity for effective, ‘anti-biofilm’ environmental cleaning and hand hygiene in infection control [43].

### A call for increased awareness

Although the implications of biofilm in chronic infections has been acknowledged since the 1980s, its involvement in antibiotic resistance has been largely overlooked. Biofilm must be considered synonymously with antibiotic resistance because of its proficiency in transferring resistance genes as well as its innate phenotypic tolerance to antibiotics. Although biofilm falls outside of the current definition of antimicrobial stewardship, greater awareness of the existence, ubiquity, and consequences of environmental biofilm amongst healthcare practitioners is crucial to improving hygiene practices and controlling the emergence and spread of antibiotic resistance in healthcare facilities.
